# Nucleoid Associated Proteins: The Small Organizers That Help to Cope With Stress

**DOI:** 10.3389/fmicb.2020.00590

**Published:** 2020-04-08

**Authors:** Joanna Hołówka, Jolanta Zakrzewska-Czerwińska

**Affiliations:** Department of Molecular Microbiology, Faculty of Biotechnology, University of Wrocław, Wrocław, Poland

**Keywords:** stress response, nucleoid associated proteins, bacterial chromosome dynamics, bacterial chromosome compaction, host survival

## Abstract

The bacterial chromosome must be efficiently compacted to fit inside the small and crowded cell while remaining accessible for the protein complexes involved in replication, transcription, and DNA repair. The dynamic organization of the nucleoid is a consequence of both intracellular factors (i.e., simultaneously occurring cell processes) and extracellular factors (e.g., environmental conditions, stress agents). Recent studies have revealed that the bacterial chromosome undergoes profound topological changes under stress. Among the many DNA-binding proteins that shape the bacterial chromosome structure in response to various signals, NAPs (nucleoid associated proteins) are the most abundant. These small, basic proteins bind DNA with low specificity and can influence chromosome organization under changing environmental conditions (i.e., by coating the chromosome in response to stress) or regulate the transcription of specific genes (e.g., those involved in virulence).

## Introduction

Bacteria have developed a plethora of strategies to inhabit nearly every environment on Earth (Boor, 2006). To survive, bacteria must quickly adapt to changing environmental conditions. To date, dozens of group- or species-specific and universal adaptive mechanisms have been uncovered (Anderson and Kendall, 2017; Singh, 2017; Bussi and Gutierrez, 2019). Among them, changes in the architecture of the entire chromosome or particular chromosome regions (e.g., gene promoters) appear to be the most rapid and effective adaptation strategies, particularly in response to sudden stress (Boor, 2006; Morikawa et al., 2006; Trojanowski et al., 2019). Such a response is apparently universal, as it has been observed in many of the bacterial species investigated to date.

To fit the bacterial chromosome along with all associated proteins and RNA inside a tiny cell, the DNA has to be compacted more than 1000-fold (Murphy and Zimmerman, 1995). The nucleoid exhibits a multi-level hierarchical structural organization similar to that of eukaryotic chromatin (Macvanin and Adhya, 2012; Badrinarayanan et al., 2015; Verma et al., 2019; Dame et al., 2020). In the model organism, *Escherichia coli*, the 4.6-Mb chromosome is organized into four structural macrodomains (Ori, Ter, Left, and Right chromosomal arms) and the two unstructured regions, each of which consists of small (average ∼10 kb) topologically independent microdomains (Postow et al., 2004; Valens et al., 2004; Espeli et al., 2008). This hierarchical structure maintains the global nucleoid organization and ensures the accessibility of particular chromosomal regions for DNA-dependent processes, such as replication, transcription, DNA repair, and recombination. The organization of the highly compacted yet dynamic nucleoid structure reflects the input of many different factors, including molecular crowding, depletion forces, DNA supercoiling, and nucleoid-associated proteins (NAPs) (Luijsterburg et al., 2006; de Vries, 2010; Dillon and Dorman, 2010; Jeon et al., 2017; Joyeux, 2019). The NAPs are small basic proteins that help compact the DNA into microdomains and also act as global regulators of transcription (Shahul Hameed et al., 2019). A great deal of studies indicated that NAPs play crucial roles in the ability of a bacterium to adapt to unfavorable conditions, particularly stress (Atlung and Ingmer, 1997; Nguyen et al., 2009; Kahramanoglou et al., 2011; Mangan et al., 2011; Datta et al., 2019). Under stress conditions, some NAPs can function as “rapid reaction forces” by introducing DNA topology changes that protect DNA or alter the transcriptional profile, particularly with respect to genes that are crucial for bacterial survival.

Here, we provide a mini review of the NAP-mediated rapid adaptation strategies that bacteria use to endure unfavorable conditions.

## Nucleoid Dynamics Are Orchestrated by NAPs

**FIGURE 1:**
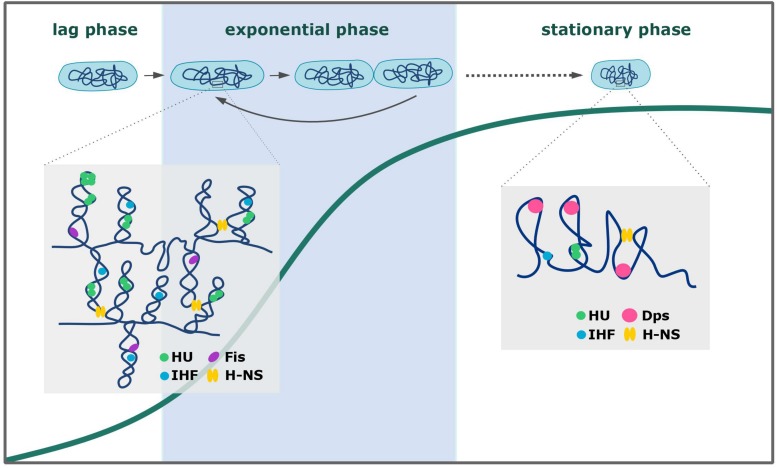
Chromosome organization during the growth of *Escherichia coli*. The expression patterns of *E. coli* NAPs reflect the chromosome compaction level (higher in the stationary than in the exponential phase) and cellular processes that involve certain NAPs (Ali Azam et al., 1999; Luijsterburg et al., 2006; Dillon and Dorman, 2010). See text for a detailed description.

The proper balance between chromosome compaction and the availability of chromosomal regions for the protein complexes involved in different cellular processes depends mainly on the DNA binding activity of NAPs (Krogh et al., 2018; Flores-Ríos et al., 2019). These small basic proteins can condense chromosomal DNA by bending, wrapping, and/or bridging relatively distant DNA strands (Luijsterburg et al., 2006; Dillon and Dorman, 2010). They all possess dimerization/oligomerization domains that facilitate chromosome coating and binding within the chromosomal regions to create inflexible filaments. Most NAPs show rather low sequence specificity for binding; however, their binding sites are often AT-rich, which is a characteristic feature of gene promoters (Kahramanoglou et al., 2011; Prieto et al., 2012; Odermatt et al., 2018). All bacterial species possess NAPs, some of which are unique for a given genus and/or species (Datta et al., 2019; Gehrke et al., 2019; Liu et al., 2019). The NAPs of *E. coli* are the best studied examples (Wold et al., 1996; Ali Azam et al., 1999; Ryan et al., 2002; Dillon and Dorman, 2010; Verma et al., 2019). The main NAPs include HU (heat-unstable protein), IHF (integration host factor), H-NS (histone-like nucleoid structuring protein), Lrp (leucine-responsive regulatory protein), Fis (factor for inversion stimulation), and Dps (DNA-binding protein from starved cells) (Luijsterburg et al., 2006; Wang et al., 2011). These NAPs can be divided based on their DNA-binding modes (Figure 1): HU, IHF, Fis, and Dps organize the chromosome by inducing bends into the DNA; H-NS can bridge two DNA strands; and in the case of Lrp, DNA is wrapped around the protein complex, enabling the joining of distant DNA strands. These DNA-binding activities of NAPs induce both topological and structural changes in the chromosomal DNA to ensure its proper compaction inside the cell. In addition to their architectural roles, NAPs are also involved in cellular processes, such as transcription (H-NS), DNA replication (HU, IHF, Fis), and DNA recombination, repair, and SOS response (HU) (Wold et al., 1996; Atlung and Ingmer, 1997; Kamashev and Rouviere-Yaniv, 2000; Ryan et al., 2002; Shahul Hameed et al., 2019). Given the variety of the functions overseen by NAPs, it is unsurprising that their expression pattern differs during growth (see Figure 1; Ali Azam et al., 1999; Dillon and Dorman, 2010; Verma et al., 2019). During the exponential phase of growth, the most abundant NAPs in *E. coli* include HU and Fis (Wold et al., 1996; Ryan et al., 2002; Kivisaar, 2020). Cells in the stationary phase produce NAPs that can most effectively condense the chromosome (e.g., Dps) (Calhoun and Kwon, 2011; Sato et al., 2013). Some NAPs (e.g., H-NS) are consistently expressed at a relatively low level, rendering them available to alter the expression of certain genes under a given stimulus (Shahul Hameed et al., 2019). NAPs have been shown to change the transcriptional profile of the cell (Atlung and Ingmer, 1997; Kahramanoglou et al., 2011), and this reportedly reflects their DNA-binding preferences. Recent studies have shown that, in addition to their growth-phase-dependent expression, some NAPs undergo posttranslational modifications (e.g., phosphorylation, acetylation, pupylation, succinylation) (Gupta et al., 2014; Ghosh et al., 2016; Okanishi et al., 2017; Dilweg and Dame, 2018). Acetylation and phosphorylation of basic residues (particularly those within the DNA-binding domain) will tend to neutralize or negatively shift the overall protein charge, respectively, which in turn decreases the DNA-binding activity of the modified NAP. Such additional control could be essential in the case of stress conditions, when the binding patterns of certain NAPs must be changed (Dilweg and Dame, 2018). The variety of NAPs and their balanced expression and activity regulation ensure the availability of chromosomal regions involved in cellular processes and enable the cell to adapt to various environmental and stress conditions. A rapid reaction to stress, which is crucial for the cell’s ability to survive, mostly relies on NAPs DNA binding activity. By influencing gene expression and/or coating the chromosomal DNA, NAPs help the cell quickly react to changing conditions and thereby protect the DNA from damage.

## NAPs Exhibit Nucleoid-Protecting Activity Under Stress Conditions

**FIGURE 2:**
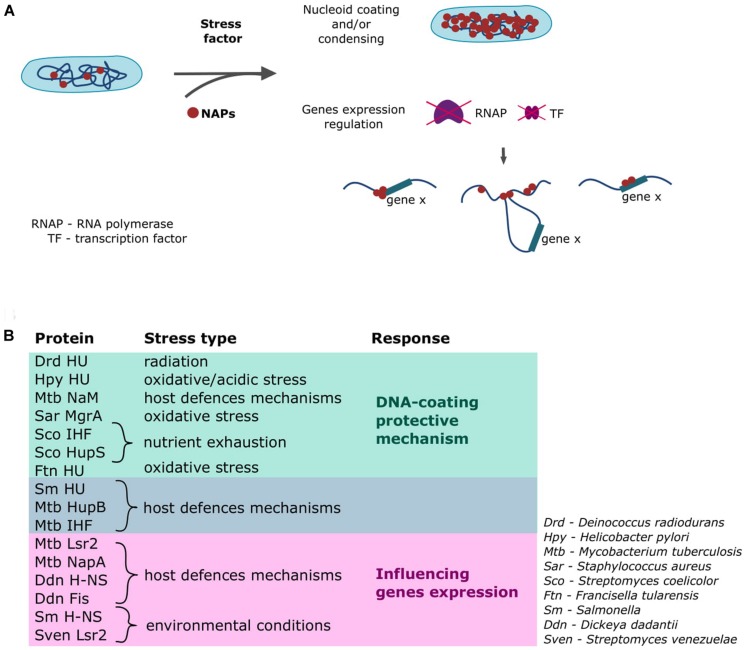
Involvement of NAPs in stress responses. **(A)** General mechanisms through which NAPs act in response to a stress factor (Dillon and Dorman, 2010; Meyer and Grainger, 2013; Kriel et al., 2018; Trojanowski et al., 2019). **(B)** Examples of the homologs of the canonical *E. coli* NAPs involved in the cellular response triggered upon detection of stress conditions.

Bacteria have developed numerous mechanisms to mount stress responses that enable the cell to adjust to changing conditions in various habitats (Boor, 2006; Bleuven and Landry, 2016). Saprophytic species living in soil or water are constantly subjected to potentially stressful environmental conditions, such as UV radiation, cold shock, heat shock, drying, and nutrient limitation. Some species survive by forming spores or endospores that can start a new population in a different niche and/or under more favorable conditions. Pathogens, meanwhile, have developed many sophisticated mechanisms that enable them to live inside the host cells (e.g., *Mycobacterium tuberculosis*, an etiological agent of tuberculosis, can survive within host alveolar macrophages for decades) (Bussi and Gutierrez, 2019). Most pathogenic species must face stress factors that reflect the host defenses mechanisms, such as low pH, oxidative stress, hypoxia, and limited nutrient availability (Anderson and Kendall, 2017; Singh, 2017; Bussi and Gutierrez, 2019; Datta et al., 2019). Beyond the systems that specifically cope with stress (e.g., the general stress response involving alternative sigma factors, the stringent response), the immediate protection comes from the NAPs (see Figure 2; Boor, 2006; Boutte and Crosson, 2013).

The cellular response will vary depending on the level, type, and duration of the perceived stress; such a response might range from the activation of precise mechanisms to the initiation of a global protective reaction that involves the whole nucleoid (Figure 2A; Atlung and Ingmer, 1997; Morikawa et al., 2006; Sato et al., 2013; Gehrke et al., 2019). The *M. smegmatis* nucleoid shrinks upon antibiotic treatment; this preserves the structure and integrity of the nucleoid and allows the cell to revive after the inhibitor removal (Trojanowski et al., 2019). Such tight chromosome compaction is also observed in the transition to the stationary phase, when the cells are shorter and there is much less room for the nucleoid (Meyer and Grainger, 2013). Some NAP family proteins can coat the whole chromosome; for example, HU can bind along the whole chromosome, although it prefers AT-rich regions and certain DNA structures (e.g., Holliday junctions, replication forks) rather than specific motifs (Kamashev and Rouviere-Yaniv, 2000; Bahloul et al., 2001). *Deinococcus radiodurans* is an extremophilic organism that is highly resistant to radiation of any type (e.g., ionizing radiation, UV light) (Makarova et al., 2001). Its genome encodes three HU protein homologs that contribute to the survival of this bacterium in unfavorable conditions (Nguyen et al., 2009). The homolog of *E. coli* HU protein encoded in the genome of pathogenic *Helicobacter pylori* also shows protective activity toward the chromosomal DNA, and a mutant strain lacking this HU-like protein exhibits increased sensitivity to oxidative and acid stress and decreased survival inside macrophages (Wang et al., 2012). The *E. coli* IHF protein shows a DNA-binding profile similar to that of HU (Azam et al., 2000; Wang et al., 2011). Both proteins exhibit a preference for AT-rich regions, but unlike HU, the IHF protein specifically recognizes 13-bp sequences with the consensus 5’-WATCAANNNNTTR-3’ (Hales et al., 1994; Prieto et al., 2012). *M. tuberculosis* possesses homologs of both HU and IHF (HupB and mIHF, respectively), and these proteins are essential during the infection of macrophages (Pandey et al., 2014; Odermatt et al., 2018). Moreover, it was shown that expression of *hupB* gene increases during the infection (Kumar et al., 2011). When faced with nutrient exhaustion in their habitat, some bacteria, such as *Streptomyces*, form spores that enable them to survive. Many agents are involved in the proper switching of the life cycle; among them, HU and IHF play vital roles. In *S. coelicolor*, sIHF (IHF homolog) and HupS (HU-like protein) are required to enable the DNA to fit inside the tiny spores (spores deprived of sIHF or HupS are temperature sensitive) (Salerno et al., 2009; Swiercz et al., 2013). An HU-like protein found in the human pathogen, *Francisella tularensis* (the causative agent of tularemia), protects the DNA against free hydroxyl radicals (Stojkova et al., 2018). A similar mechanism of action is exhibited by the *Staphylococcus aureus* MgrA protein; this homolog of *E. coli* Dps coats the DNA, protecting it against oxidative stress and ensuring prolonged survival of the cell inside phagosomes (Crosby et al., 2016; Ushijima et al., 2017). It has been reported that the *M. tuberculosis* genome encodes a novel NAP, called NapM, that is also required for the pathogen to survive inside the host macrophages (Liu et al., 2019). The NapM sequence homolog from *M. smegmatis* was shown to colocalize with the *E. coli* nucleoid (Liu et al., 2016), potentially suggesting that this protein exhibits similar binding modes in pathogenic mycobacteria.

The NAPs involved in the protective DNA-coating mechanism share a few similarities, including a relatively low DNA-binding specificity and a high copy number (Ali Azam et al., 1999; Dillon and Dorman, 2010; Verma et al., 2019). Homologs of the canonical “whole-chromosome binders” (i.e., HU, IHF, Dps) often possess unique structural features that ensure their effective binding along the entire chromosome. HU-like proteins in Actinobacteria (e.g., mycobacterial HupB, *S. coelicolor* HupS) have additional positively charged C-terminal domains that have been shown to stabilize the DNA-protein complexes (Salerno et al., 2009; Hołówka et al., 2017). *D. radiodurans* HU homologs have also repetitive basic residues, but within the N-terminal domain (Ghosh and Grove, 2006). The DNA-coating mechanism is activated immediately when unfavorable conditions are sensed, and helps maintain chromosomal integrity by creating a physical barrier against stress factors, such as radiation, antibiotic treatment, oxidative and acidic stress.

## NAPs Alter Basic Cellular Processes in Response to Stress

The abilities to mount a rapid and effective response to changing environmental conditions and/or adjust the cell’s metabolic activity to prolonged stress are key factors in the survival of both pathogens and saprophytes. The most “specific” stress response mechanism involving NAPs relies on their ability to influence the expression level of the certain gene(s) and/or gene cluster(s) (Kahramanoglou et al., 2011; Kriel et al., 2018; Flores-Ríos et al., 2019; Dorman et al., 2020). These small nucleoid organizers can affect transcription by inducing topological and/or structural changes in the chromosomal DNA (Figure 2A) that can alter the binding of RNA polymerase or transcription factors. Moreover, as mentioned earlier, NAPs often bind to AT-rich regions within promoter sequences and thereby repress gene expression. Depending on a given NAP’s DNA-binding specificity and number of target DNA sequences, it can simultaneously affect the transcription of many genes/gene clusters or act as a specific switch that alters the expression levels of certain genes (Gordon et al., 2010; Prieto et al., 2012; Gehrke et al., 2019; Shahul Hameed et al., 2019). H-NS, which exhibits DNA-bridging activity (Figure 1), was shown to be a global transcription repressor in human pathogens, including *Salmonella enterica serovar Typhimurium*, *Vibrio cholerae*, and toxigenic strains of *E. coli* (Ayala et al., 2015; Helgesen et al., 2016; Shahul Hameed et al., 2019). Additionally, it was shown that a H-NS paralog, StpA protein cooperate with H-NS to alter virulence genes expression in uropathogenic *E. coli* strains (Müller et al., 2006). The *E. coli* H-NS binding sites are reportedly clustered near the *ter* region, where genes connected with motility and biofilm formation are localized. Interestingly, H-NS from *Salmonella* acts as a repressor for horizontally acquired pathogenicity islands (Lucchini et al., 2006; Brunet et al., 2015). Similarly, a structural homolog of H-NS in *M. tuberculosis* (called Lsr2) is involved in the regulation of many genes, including those connected with virulence (Gordon et al., 2010). Deletion of the *lsr2* gene results in decreased growth and survival under hypoxia (Bartek et al., 2014), suggesting that Lsr2 could be a crucial agent that “switches” mycobacteria to the dormant state and enables them to endure inside host cells. The recently described NapA protein is another mycobacterial NAP that serves as a global transcription factor (Datta et al., 2019). It exhibits a preference for AT-rich regions and coats the DNA to create inflexible rods that interrupt DNA supercoiling. *M. tuberculosis* NapA regulates the expression of genes that encode virulence regulators. An Lsr2-like protein produced by another member of Actinobacteria, the saprophytic *S. venezuelae*, was shown to control genes whose products are involved in signaling and producing specialized secondary metabolites (Gehrke et al., 2019). Homologs of the *E. coli* H-NS and Fis proteins produced in the plant pathogen, *Dickeya dadantii*, influence the expression levels of the *pal* genes, which act as major virulence factors (Ouafa et al., 2012). Intriguingly, HU-like proteins found in *Salmonella* and *F. tularensis* not only create the physical protective barrier against stress factors, they also regulate genes involved in general physiology, metabolism, and virulence (Figure 2B; Mangan et al., 2011; Stojkova et al., 2018). The mycobacterial HupB protein regulates the expression of the *katG* gene (acting as a repressor), whose product activates the anti-tuberculosis drug, isoniazid (Niki et al., 2012; Enany et al., 2017); a *M. smegmatis* strain deprived of HupB showed increased susceptibility to this drug (Hołówka et al., 2017). Additionally, recent studies showed that the *M. tuberculosis* mIHF protein represses the expression of many genes, including those connected with pathogenesis (Odermatt et al., 2018).

In addition to their conventional architectural role and involvement in regulating gene expression, NAPs may also contribute to other cellular processes. For example, studies have shown that NAPs influence chromosome replication by binding and inducing some structural changes within the origin of chromosomal replication (*oriC*). In *E. coli*, the IHF and HU proteins facilitate the formation of the pre-replication complex, and the Fis protein prevents replication initiation (Wold et al., 1996; Ryan et al., 2002). Interestingly, expression of the *M. tuberculosis napM* gene increases upon stress; the NapM protein binds DnaA (a replication initiation protein) to inhibit chromosome replication, which in turn ensures that mycobacteria transition to the dormant state to survive inside host macrophages (Liu et al., 2019). Almost all processes involving spatial transitions of DNA strands, such as DNA repair and recombination and the topoisomerase-mediated maintenance of topological homeostasis, are based on cooperation with NAPs (e.g., HU interacts directly with topoisomerase A to alter its DNA-relaxing activity) (Shanado et al., 1998; Kamashev and Rouviere-Yaniv, 2000; Ghosh et al., 2014; Kivisaar, 2020). Overall, the low DNA-binding specificity and relatively high copy number of NAPs make them readily available and able to assist with complex cellular processes. Proper synchronization of the processes occurring inside the cell with constantly changing environmental conditions is a key element to survival under stress.

## Conclusion

During the course of their evolution, bacteria developed the ability to rapidly adapt to constantly changing environmental conditions. Rapid reactions to many different signals, including stress factors, are crucial for the survival of both saprophytes and pathogens. As reviewed herein, NAPs ensure the very efficient and immediate response to various stimuli. These small basic proteins shape chromosomal DNA, adjusting its architecture in response to intra- and extracellular conditions. When the bacterial cell detects strong stress, NAPs (e.g., HU, Dps) generally coat and/or condense the nucleoid, creating a physical protective barrier for the DNA (Nguyen et al., 2009; Salerno et al., 2009; Pandey et al., 2014; Crosby et al., 2016; Odermatt et al., 2018). More specific NAP-related stress response mechanisms involve the ability of NAPs to regulate transcription. Upon binding, NAPs (e.g., H-NS, Fis) induce structural and/or topological DNA changes that lead to alteration of the expression levels of certain genes (Kahramanoglou et al., 2011; Brunet et al., 2015). Many genes involved in the adaptation to a new living condition, such as by formation of biofilm or alteration of motility, synthesis of secondary metabolites, and/or virulence, are regulated by NAPs. Additionally, NAPs can regulate basic cellular processes (e.g., replication initiation) in order to synchronize such processes with changing environmental conditions (Ryan et al., 2002; Datta et al., 2019). Hence, most NAPs act as the “rapid reaction forces” that enable the bacterial cell to endure under stress.

## Author Contributions

JH wrote the main body of the manuscript, conclusion and prepared figures. JZ-C wrote the introduction and revise the entire manuscript.

## Conflict of Interest

The authors declare that the research was conducted in the absence of any commercial or financial relationships that could be construed as a potential conflict of interest.
